# Disrupted sleep without sleep curtailment induces sleepiness and cognitive dysfunction via the tumor necrosis factor-α pathway

**DOI:** 10.1186/1742-2094-9-91

**Published:** 2012-05-11

**Authors:** Vijay Ramesh, Deepti Nair, Shelley X L Zhang, Fahed Hakim, Navita Kaushal, Foaz Kayali, Yang Wang, Richard C Li, Alba Carreras, David Gozal

**Affiliations:** 1 Section of Sleep Medicine, Department of Pediatrics, Pritzker School of Medicine, The University of Chicago, Chicago, IL, USA; 2 Department of Pediatrics, The University of Chicago, 5721S. Maryland Avenue, MC 8000, Suite K-160, Chicago, IL, 60637, USA

**Keywords:** TNF-α, Sleep fragmentation, Neurocognitive impairments, Sleep apnea, ATP

## Abstract

**Background:**

Sleepiness and cognitive dysfunction are recognized as prominent consequences of sleep deprivation. Experimentally induced short-term sleep fragmentation, even in the absence of any reductions in total sleep duration, will lead to the emergence of excessive daytime sleepiness and cognitive impairments in humans. Tumor necrosis factor (TNF)-α has important regulatory effects on sleep, and seems to play a role in the occurrence of excessive daytime sleepiness in children who have disrupted sleep as a result of obstructive sleep apnea, a condition associated with prominent sleep fragmentation. The aim of this study was to examine role of the TNF-α pathway after long-term sleep fragmentation in mice.

**Methods:**

The effect of chronic sleep fragmentation during the sleep-predominant period on sleep architecture, sleep latency, cognitive function, behavior, and inflammatory markers was assessed in C57BL/6 J and in mice lacking the TNF-α receptor (double knockout mice). In addition, we also assessed the above parameters in C57BL/6 J mice after injection of a TNF-α neutralizing antibody.

**Results:**

Mice subjected to chronic sleep fragmentation had preserved sleep duration, sleep state distribution, and cumulative delta frequency power, but also exhibited excessive sleepiness, altered cognitive abilities and mood correlates, reduced cyclic AMP response element-binding protein phosphorylation and transcriptional activity, and increased phosphodiesterase-4 expression, in the absence of AMP kinase-α phosphorylation and ATP changes. Selective increases in cortical expression of TNF-α primarily circumscribed to neurons emerged. Consequently, sleepiness and cognitive dysfunction were absent in TNF-α double receptor knockout mice subjected to sleep fragmentation, and similarly, treatment with a TNF-α neutralizing antibody abrogated sleep fragmentation-induced learning deficits and increases in sleep propensity.

**Conclusions:**

Taken together, our findings show that recurrent arousals during sleep, as happens during sleep apnea, induce excessive sleepiness via activation of inflammatory mechanisms, and more specifically TNF-α-dependent pathways, despite preserved sleep duration.

## Background

Sleep fragmentation (SF), unlike prolonged sleep deprivation, is a common consequence of many sleep disorders in humans [1,2], of narcolepsy [3], and of suboptimal sleeping conditions, such as noisy environments. A minimum period of uninterrupted sleep is essential for optimal daytime vigilance and neurocognitive and behavioral functions [4-6]. Cytokines such as tumor necrosis factor (TNF)-α and interleukin (IL)-1beta are multifunctional pro-inflammatory cytokines, which have been recognized not only as crucial inflammatory mediators, but also as important mechanisms involved in the regulation of sleep[7], aging and neurodegenerative diseases associated with aging [8,9], and learning[10,11]. TNF-α can be synthesized and released in the brain by both neurons and glial cells, and exerts multiple functions by binding to two different TNF receptors (p55 (TNFR1) and p75 (TNFR2)), which are constitutively expressed in the nervous system [12]. TNF-α and IL-1β enhance slow wave sleep (SWS), and inhibition of TNF-α or IL-1β reduces spontaneous sleep. Exogenous injection of TNF-α or IL-1β into animals and/or humans induces sleepiness and elicits excess sleep [7], whereas prolonged wakefulness upregulates both TNF-α and IL-1β in the brain [7]. Interestingly, pathological concentrations of TNF-α inhibit long-term potentiation (LTP), a surrogate reporter of learning and memory in the hippocampus [12-16], and impair cognitive function [17].

In the brain, expression of Homer1a is increased after sleep loss, suggesting a role for sleep in the regulation of intracellular calcium homeostasis, particularly in protection and recovery from the calcium-pool changes induced by the prolonged neuronal activation imposed by extended wakefulness [18]. According to Tononi and Cirelli, the plastic processes occurring during wakefulness result in increased synaptic strength, whereas the role of sleep is to downscale synaptic strength to a basal level while the newly acquired information is retained. Both environmental and pharmacological stressors upregulate Homer1a mRNA in key structures involving higher brain functions [19]. Spontaneous wakefulness has been shown to be associated with the diffuse induction of molecular changes usually associated with LTP [20,21], including the phosphorylation of cyclic AMP response element-binding protein (CREB) and the induction of genes such as Arc, brain-derived neurotrophic factor, nerve growth factor-induced gene A , Homer, and neuronal activity-regulated pentraxin [22-24]. Type 4 cAMP phosphodiesterase (PDE)4, a PDE enzyme that hydrolyzes cAMP, is known to play an important role in memory processes. PDE4 inhibition increases intracellular availability of cAMP, which is known to activate the downstream target CREB protein after activation of protein kinase A. This signaling cascade is important in the consolidation of memory processes and synaptic plasticity [25-27]. Furthermore, rolipram, a selective inhibitor of PDE4, was shown to completely reverse the amnesic effects of MK-801 on working and reference memory [28] via increased cAMP/CREB signaling in the hippocampus [29]. The sensory, motor, or cognitive activities that occur during active wakefulness are often associated, in a small subset of neurons, with high peak firing rates that are likely to give rise to LTP-related plastic changes [30]. This induction of LTP-related genes during spontaneous wakefulness can increase further if animals are kept awake longer by gentle handling, or if they engage in extensive exploration of their environment [31]. By contrast, during sleep the expression of LTP-related genes is severely reduced or abolished [20,21,32]. Support for the notion that synaptic strength may increase during wakefulness also comes from experiments in humans [33]and mice [34,35] showing that brain metabolism, which is mostly due to synaptic activity, increases from early to late wakefulness.

SF is one of the hallmark characteristics of sleep apnea. Experimentally induced short-term SF, even in the absence of any reductions in total sleep duration, will lead to the emergence of excessive daytime sleepiness and cognitive impairments in humans [4,6]. However, the vast majority of the studies aiming to unravel the role of sleep in the homeostatic regulation of biological systems has focused on sleep deprivation [36-38], or alternatively has used SF procedures only for short periods [39-42]. To examine the effects of long-term SF in mice, thereby mimicking the long-standing clinical course of sleep apnea preceding its diagnosis, we took advantage of a newly designed and validated device that does not require increments in locomotion, precludes the need for tethering or social isolation, and is not associated with increase in corticosterone levels [43].

## Methods

The experimental protocols were approved by the Institutional Animal Use and Care Committee and are in close agreement with the National Institutes of Health Guide in the Care and Use of Animals. All efforts were made to minimize animal suffering and to reduce the number of animals used.

### Animals

Male C57BL/6 J mice TNF-α receptor double knockout mice (TNFR-KO; catalog #003243, B6;129 S-Tnfrsf1atm1Imx Tnfrsf1btm1Imx/J; Jackson Laboratories, Bar Harbor, ME, USA), matched for age and weight (20–25 g), were housed with a 12 hour light/dark cycle (07.00 to 19.00 hours) at a constant temperature (26 ±1°C), and allowed access to food and water *ad libitum*.

### Sleep fragmentation exposures

The SF device used to induce SF in rodents has been previously described [43] (catalog # Model 80390, Lafayette Instruments, Lafayette, IN, USA), and employs intermittent tactile stimulation of freely behaving mice in a standard mouse laboratory cage, using a near-silent motorized mechanical device. All SF procedures were carried out for 12 hours from 07.00 to 19.00 hours during the light period (LP).

### Surgical procedure and implantation of telemetric transmitter and electrodes

All surgical procedures were performed under sterile conditions and general anesthesia. A telemetric transmitter weighing 3.5 g (F20-EET; DSI, MN, USA), which allows simultaneous monitoring of two biopotential channels (temperature and locomotor activity) were chronically implanted to record an electrencephalogram (EEG) from the frontal area and an electromyogram (EMG) from the superior nuchal muscle [43] .

Sleep recordings, SF, and sleep scoring were performed as previously described [43,44]. Behavior was classified into three different states: wake, SWS, and rapid eye movement (REM) sleep. EEG during wake had low-amplitude, high-frequency (desynchronized) waves. During wake, EMG records showed gross body movement artifacts, and behaviorally, animals had grooming, scratching, and orienting activity. The SWS stage was characterized by low-frequency, high-amplitude (synchronized) EEG with a considerable reduction in EMG amplitude. REM sleep was characterized by desynchronized EEG, and a drastic reduction in EMG (muscle atonia). Sleep-related low frequency (delta) activity was also derived from the records using bandpass filtering of 1 to 4.0 Hz. Delta power was computed offline by Fast Fourier Transform (FFT) using SleepSign software, Kissei Comtec Co., LTD., Nagano, Japan which was based on 512 points corresponding to 10 second epochs, at a sampling rate of 250 Hz, with Hanning as the window filter of FFT. The software algorithm analysis was based on The Nyquist–Shannon sampling theorem, which states that perfect reconstruction of a signal is possible when the sampling frequency is greater than twice the maximum frequency of the signal being sampled. Those SWS epochs that showed movement artifacts were excluded when computing delta power, because EEG signals are especially sensitive to movement, with the resulting artifact specifically enhancing signals in the delta band. SWS latency was calculated as the time taken from each arousal episode to the first epoch of SWS.

For the neutralizing antibody studies (TNF-α antibody (Ab) + SF), we gave the C57BL/6 J mice an intraperitoneal injection of either vehicle (saline) or TNF-neutralizing antibody (clone TN3-19; eBiosciences, 100 μg per mouse/day) 10 minutes before starting the SF procedures) [45].

### Behavioral testing

The Morris water maze was used to assess spatial reference learning and memory, and working memory. The maze protocol is similar to that described by Morris [46], with modifications for mice. Briefly, a standard place-training reference memory task was conducted on mice in the water maze after exposure to 15 days of SF or saline. Place learning was then assessed over six consecutive days using a spaced training regimen that has been found to produce optimal learning in mice [47]. Maze performance was recorded by a video camera suspended above the maze and interfaced with a video tracking system (HVS Imaging, Hampton, London, UK). The performance in the water maze was assessed using mean escape latencies and swim distance. Retention tests were carried out 15 days after acquisition of the task as previously described [48]. For the elevated plus maze (EPM), which is a measure of anxiety, mice were placed in the center of the maze facing a closed arm, and allowed to explore for 10 minutes in isolation. Data acquisition and analysis were automatically processed (Noldus Ethovision Software; Leesburg, VA). The percentage time spent in the open, the number of entries into the closed arms, and the time spent in the center were analyzed. In the forced swimming test (FST), a measure of murine depression and anhedonia, mice were individually forced to swim for 6 minutes on two consecutive days in an open cylindrical container (diameter 140 mm; temperature 25 ± 1°C). Mice were marked as immobile if they performed the minimal amount of work required to float for at least 1 second as previously described [49-51]. Of note, the mice were returned to the SF cages immediately after the behavior tests (<60 minutes), so that there was no recovery phase in these mice.

### Measurement of ATP levels

Cortical tissues were snap-frozen in liquid N_2_ within 20 seconds and were pulverized on LN2 for nuclear acid extraction using ice-cold 5% trichloroacetic acid (TCA). The mixture was sonicated with a probe sonicator for 10 pulses and separated by centrifugation at 10,000 *g* and 4°C for 15 minutes. The supernatant was collected and neutralized with Tris buffer (1 mol/L, pH 7.8). ATP levels were then measured using a commercial kit )ATP Bioluminescence Assay Kit CLS II; Roche Applied Science, Indianapolis, IN, USA). Protein concentrations in the supernatant were determined using a protein assay kit (Bio-Rad Dc; Bio-Rad, Hercules, CA, USA) to normalize ATP levels.

### Assessment of AMP kinase α activation

Snap-frozen cortical tissues were pulverized on liquid N_2_ and immediately homogenized in 1% SDS preheated to 92°C. The homogenate was separated by centrifugation at 14,000 *g* for 15 minutes, and the supernatant containing total cellular proteins was collected. This method has been shown previously to effectively inhibit activation of phosphorylation and dephosphorylation processes associated with many routine protein preparation protocols [52,53]. The protein concentration was determined (Microplate BCA Protein Assay Kit; Pierce Biotechnology, Rockford, IL, USA) and samples were then subjected to western blotting analysis for AMP-activated protein kinase (AMPK) activation. The same blot was used for detection of the phospho-AMPK-α (anti-pAMPKα; Cell Signaling Technology, Danvers, MA, USA) and the total AMPK-α (anti-AMPKα; Cell Signaling Technology) and positive signals were visualized with enhanced chemiluminescence.

### CREB DNA binding assay

Nuclear extracts from hippocampal tissues harvested from mice exposed to SF or control sleep conditions were prepared (Nuclear Extract Kit; cat. # 40010; Active Motif, Carlsbad CA, USA), in accordance with the manufacturer’s instructions, using a dounce homogenizer. For the DNA binding assays, six animals per condition were studied. CREB nuclear binding was assessed using (Trans-AM phospho-CREB activation Assay Kit; cat. #43096; Active Motif, Carlsbad, CA). This enzyme-linked immunosorbent assay employs an antibody that selectively recognizes the phosphorylated epitope of CREB when the latter is specifically bound to a specific oligonucleotide coated onto a 96-well plate. Specificity of the binding was further monitored by competition with free wild-type and mutated oligonucleotides. Samples were assessed by a spectrophotometer at 450 nm.

### Quantitative real-time PCR

The mRNA expression (TNF-α, IL-1, IL-6, TNFR1, TNFR2, and phosphodiesterase 4) was determined by quantitative RT-PCR using commercially available specific primers.

### TNF-α ELISA

TNF-α brain cortical levels were measured in triplicate using a commercially available ELISA assay in accordance with the manufacturer’s instructions (Mouse TNF-alpha ELISA Kit, OptEIA™; BD Biosciences). This method has a minimum detection level of 0.25 pg/ml with intra-assay and inter-assay coefficients of variability of 7.4% and 7.8% respectively, and a dynamic linear range of 2.2 to 2,500 pg/ml. Assays were deemed acceptable if triplicate values were within 10% of each other.

### Immunohistochemistry

Anesthetized mice were perfused transcardially with 0.9% NaCl followed by 4% formalin, then the brains were removed, fixed in 4% formalin, and cryoprotected with 30% sucrose. Coronal sections (40 um) were incubated in 0.3% H_2_O_2_ for 30 minutes, washed in PBS, and blocked with a buffer of PBS containing 0.4% Triton X-100, 0.5% tyramide signal amplification (TSA) blocking reagent (New England Nuclear Life Science Products, Boston, MA, USA) containing 10% normal goat serum (NGS; Vector Laboratories, Burlingame, CA, USA) for 1 hour. Sections were then serially incubated with either TNF-α (cat # RM90115 1:1,000; Invitrogen Corp., Carlsbad, CA, USA) or pCREB antibody (1:1000; Cell Signaling Technology, Beverly, MA, USA) at 40 C for 48 hours with a biotinylated anti-rabbit antibody (1:600 in the PBS/0.5% TSA/10% NGS blocking reagent described above) (Vectastain Elite ABC kit, Vector Laboratories), and then with streptavidin-horseradish peroxidase (diluted 1:100 in the blocking reagent as before) followed by tetramethyl rhodamine tyramide diluted 1:50 for 2 minutes (Perkin Elmer Life Sciences). Sections were subsequently incubated with serum raised against the neuronal marker Neu-N (1:1000; Chemicon, Temecula, CA), followed by a biotinylated anti-rat antibody (1:200 l Vectastain Elite ABC Kit), and by fluorescein tyramide reagent (1:50) for 3 minutes (Perkin Elmer Life Sciences). Sections were then washed in PBS and mounted onto glass slides. In general, 20 sections/animal were visualized using both fluorescent and confocal microscopes.

### Data analysis

To elucidate the nature of identified interactions between control baseline sleep characteristics and those emerging after 15 days of SF in C57BL/6 J mice and TNF-α receptor knockout mice, the data were analyzed by one-way repeated-measures ANOVA. First, overall statistical significance between the treatment groups (baseline and SF) was determined for the 24-hour period, followed by post-hoc Tukey tests, as needed. Delta power during SWS, wake episodes, and the latency of SWS after each episode of wake were also treated with similar statistical approaches. Similar statistical approaches were used to compare the entire training period between the treatment groups. In addition, two-way repeated measures ANOVA were used to analyze each trial blocks, followed by *post hoc* Tukey tests. The same statistical approaches were used to compare probe trial, reference memory, EPM, and FST. For all comparisons, *P* < 0.05 was considered significant. For behavioral test assessments, all the experimental conditions, the data were divided into six blocks (of 3 trials/day). We used a multivariate MANOVA model (SPSS software version 11; SPSS Inc., Chicago, IL, USA) that included latency, path length, and swim speed, and two between-group factors: 1) Groups (four levels): Sleep control (SC)-C57BL6/J, SC-TNF-KO and TNF-KO mice exposed to 15 days SF (SF-TNF-KO) ; and 2) condition (two levels): SC or SF. All F statistics are reported using Pillai’s Trace. The interaction of three different factors (time, condition, and group) were determined using this mixed-model repeated-measures MANOVA. The biochemical assays were analyzed using one-way ANOVA.

## Results

### Sleep architecture and behavioral changes after 15 days of sleep fragmentation in C57BL/6 J mice

#### Measurements of wake and sleep

**Figure 1:**
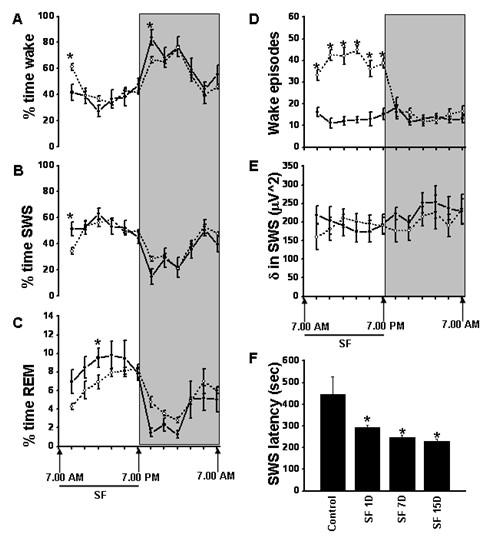
**Circadian sleep characteristics and sleep state distribution during baseline conditions (solid line) and after 15 days of sleep fragmentation (dotted line) in mice. (A–C)** There was an absence of significant alterations in sleep architecture, despite **(D)** continuing successful induction of episodic awakenings. **(E)** No changes in delta frequency power of the EEG emerged; but there were **(F)** marked reductions in mean sleep latency. (BL) baseline vs. sleep fragmentation (SF); *P < 0.0001; n = 12/group). Shaded regions represent dark phase of the circadian cycle (07.00 to 19.00 hours).

Overall analysis of the polygraphic data for a period of 24 hours (n = 12/group) showed significant differences between baseline and SF (*P* < 0.001), indicating that SF had influenced state (wake, SWS, and REM sleep). EEG monitoring during the 12-hour LP (07.00 to 19.00 hours) showed that the C57BL6/J mice were awake for 38.52 ± 4.11% of the time; after 15 days of SF, they spent 42.66 ± 0.85% of the time awake. Analysis of SWS and REM sleep during LP showed that the animals spent 52.81 ±9.90% and 8.68 ±1.73% of the time in SWS and REM sleep respectively, during baseline, which decreased to 50.39 ±0.86% and 6.9 ± 0.33%, respectively, after 15 days of SF. During the dark period (DP), there was no significant variation in wake, SWS, or REM sleep in animals subjected to 15 days SF, who had values of 58.27 ± 0.78, 36.9 ± 0.66 and 4.83 ± 0.13%, respectively, compared with baseline values of 64.59 ± 3.66, 32.01 ± 3.51 and 3.4 ± 0.35% (Figure 1A–C). There was also no change in total time spent (24 hours) in wake, SWS, and REM sleep after 15 days SF (50.46 ±0.78, 43.64 ±0.70 and 5.89 ±0.18% respectively) compared with baseline (51.55 ±3.26, 42.41 ±3.35 and 6.03 ±0.23%) (Figure 1A–C).

#### Wake episodes, delta powe,r and SWS latencies

Mice subjected to SF for 15 days maintained total sleep duration, with values comparable with the baseline values, and also globally maintained sleep state distribution and duration, despite ongoing recurring awakenings induced by the SF procedures. After 15 days of SF, there was a significant increase in wake episodes during LP (39.58 ±1.77 per 2 hours) compared with baseline (13.38 ±2.08 episodes per 2 hours) (*F* = 40.02, *P* < 0.001] indicating intermittent arousals (Figure 1D). However, there was no difference in the frequency of wake episodes during the DP after cessation of SF procedures, with 15.42 ±1.75 episodes per 2 hours. A significant increase in wake episodes after 15 days of SF was therefore recorded for the 24 hour period (mean for 24-hour period: 27.15 ±0.81 per 2 hours) compared with baseline (13.62 ±1.76 per 2 hours) (Figure 1D).

Delta power during SWS of the EEG remained unchanged after SF (192.88 ±25.09 μV^2^ at baseline vs. 189.28 ±25.85 μV^2^ after SF; Figure 1E), even though delta power is increased during sustained wakefulness, is highest during the initial cycles of SWS, decreases across the biological night, is maximally expressed in frontal derivations, and shows rebound effects after sleep deprivation, thereby accounting for its extensive use as a marker of homeostatic sleep regulation [54-56]. After cessation of SF procedures, there were no differences in delta power during the DP, and no changes in delta power emerged across 24 hours(Figure 1E). However, as previously shown [48], SF-exposed mice exhibited markedly reduced SWS latencies [57] and theta EEG frequency waking activity [58], both of which are strong indicators of the presence of excessive sleepiness (Figure 1F). Overall analysis of the data for a period of 24 hours found significant changes between baseline and SF (*P* < 0.001), indicating that SF had significant effects on SWS latency. The latency to SWS was significantly reduced throughout the LP during the SF procedures. On average, the control animals had a latency of 368.16 ±72.73 seconds to SWS at baseline, compared with 73.14 ±2.86 seconds on day 15 (*P* < 0.0001), indicating progressive increases in sleep propensity. Immediately after cessation of SF procedure, the latency to SWS during DP had a tendency to return to baseline levels. However, for a total period of 24 hours (from 0700 to 0700 hours the next day), the latency to SWS remained significantly lower after SF (230.47 ±8.88 seconds) compared with baseline (443.87 ±82.06 seconds (*F* = 18.823, *P* < 0.001) (Figure 1F). Quiet wake theta activity during the DP was significantly increased after SF (F = 15.906, *P* < 0.003).

**Figure 2:**
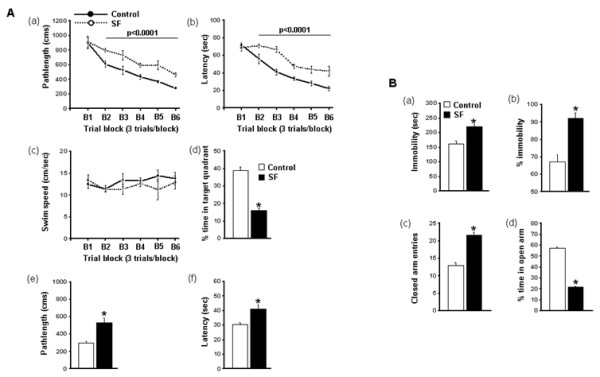
**Spatial learning performance.** (**A**) (**a, b**) Mean path lengths (cm) and latencies (seconds) to locate the target platform during place training, (**c**) swim speed and (**d**) mean percentage time in the target quadrant during probe trials after completion of water maze testing, with (**e**) path lengths and (**f**) latencies during assessment of spatial task retention in the water maze in mice exposed to 15 days of sleep fragmentation and those maintained in control sleep conditions (n = 18 per group; *P<0.0001). (**B**) Mice exposed to sleep fragmentation (**a, b**) showed less immobility during the forced swim test, (**c**) performed significantly more entries into the closed arms, and (**d**) spent significantly less time in the open arms of the elevated plus maze compared with mice having control sleep conditions (n = 18/group; * *P*<0.002).

#### Spatial learning performance

On a standard place-discrimination task, C57BL6/J mice exposed to 15 days of SF (SF-C57BL6/J) exhibited longer latencies and path-lengths to locate the hidden platform compared with control mice (SC-C57BL6/J), (n = 18 per experimental condition, Figure 2Aa, 2Ab). Overall analysis of the entire trial blocks showed significant differences between the SF and control sleep treatment groups for latency (*F* = 24.291; *P* < 0.001) and path length, (*F* = 17.785; *P* < 0.001), indicating that SF adversely affected task performance. Significant differences in latencies were seen during blocks 2 (*F* = 7.240; *P* < 0.021), 3 (*F* = 24.426; *P* < 0.001), 4 (*F* = 20.290; *P* < 0.001), 5 (*F* = 8.401; *P* < 0.014) and 6 (*F* = 7.924; *P* < 0.017). There were no significant differences in block 1. Repeated measures ANOVA showed significant differences in path lengths during blocks 2 (*F* = 10.226; *P* < 0.008), 3 (*F* = 15.25; *P* < 0.004), 4 (*F* = 14.483; *P* < 0.003), 5 (*F* = 12.496; *P* < 0.005) and 6 (*F* = 32.024; *P* < 0.001), with no significant differences in block 1. There were no significant differences in swim speed (Figure 2Ac). In the probe-trial test, one-way ANOVA showed a significant effect of treatment [SF vs. SC: *F* = 76.017; *P* < 0.001). The magnitude of impairment was greatest in SF-C57BL6/J mice (Figure 2Ad). In the reference memory tests, SF-C57BL6/J mice exhibited significant deficits in memory retention (Figure 2Ae, 2Af) in both latency (*F* = 11.662; *P* < 0.006) and path length (*F* = 17.696; *P* < 0.001).

**Figure 3:**
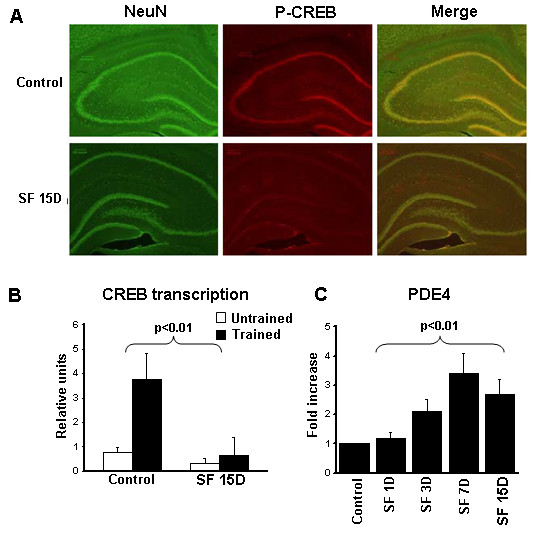
**Effects on cyclic AMP response element-binding protein (CREB). **(**A**) Phosphorylated CREB expression (red fluorescence) and NeuN(green fluorescence) in hippocampus of a representative animal exposed to sleep fragmentation (SF) for 15 days and a control (n = 3). Right panel shows merged images. (**B**) Transcriptional CREB activity in untrained mice and in mice trained in the spatial task water maze (both exposed to SF for 15 days) compared with trained and untrained non-SF controls (*P* <0.01; n = 5/group). (**C**) Time course of phosphodiesterase (PDE) 4 gene expression in cortex of mice exposed to SF (*P* <0.01 for all time points; n = 6).

#### Forced swim test

SF-C57BL6/J mice had significantly longer periods of immobility during the last 4 minutes of the FST (*F* = 24.951; *P* < 0.001) and a significantly higher percentage of time spent immobile (*F* = 22.951; *P* < 0.001) compared with the SC-C57BL6/J sleep controls (Figure 2Ba and 2Bb).

#### Elevated plus maze

There were significant differences between SF-C57BL6/J mice and controls in in the percentage of time spent in the open arm (*F* = 89.25; *P* < 0.001) and in the number of entries into the closed arm (*F* = 53.16; *P* < 0.001) (Figure 2Bc, 2Bd).

#### CREB transcription, phosphodiesterase-4 and ATP levels

**Figure 4:**
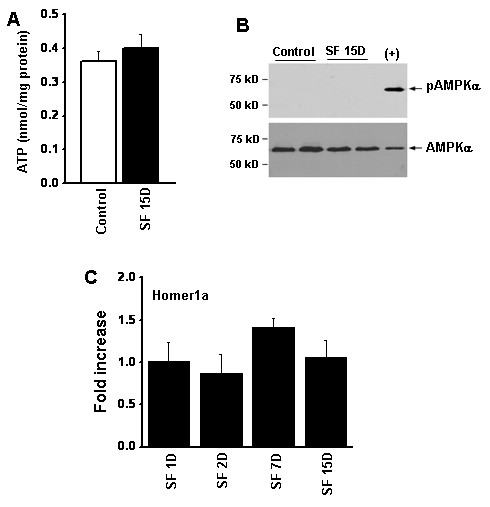
**Effect of 15 days of sleep fragmentation (SF) on energy metabolism in the cortex.** (**A**) ATP levels in cortical tissues from mice with or without SF; there was no significant difference between the groups. (n = 4/group) (**B**) Representative western blots showing the lack of -activated protein kinase (AMPK)α phosphorylation in the same tissues. E10 epithelial cells exposed to 0.2% O2 for 24 hours were used as a positive control (+) for phospho- (p)AMPK. (**C**) Homer1a expression remains unaltered over the course of SF (n = 6/time point).

The SF-induced cognitive and behavioral alterations were accompanied by reduced CREB phosphorylation in hippocampus (Figure 3A) and reduced CREB transcriptional activity. Similarly, CREB transcriptional activity in the hippocampus was markedly reduced before and after spatial task training (*P* < 0.01) (Figure 3B). Furthermore, increased expression of phosphodiesterase-4 was seen after SF (Figure 3C). However, there was no alteration in ATP levels (Figure 4A) andAMP kinase-α phosphorylation levels (Figure 4B) after SF, and Homer1a gene expression remained unchanged (Figure 4C).

**Figure 5:**
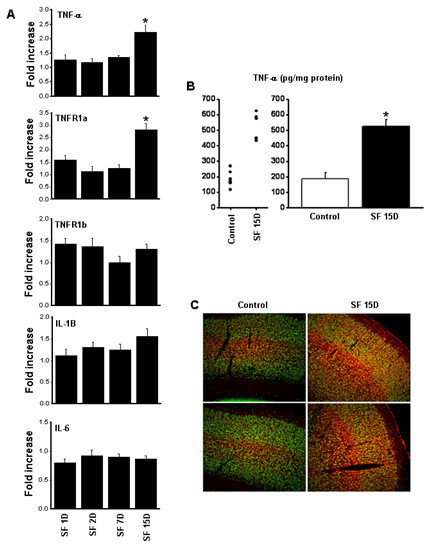
(**A) Time course of tumor necrosis factor (TNF)-α, TNF receptors, interleukin (IL)-1, and IL-6 gene expression in cortex of miceexposed to SF** (*P **<0.01; n = 6).** (**B**) TNF-α protein concentrations in cortex of mice exposed to sleep fragmentation (SF) and controls(*P <0.01). (**C**) Immunofluorescence photomicrographs in frontal cortex of two representative mice exposed to SF for 15 days and controls(n = 4), showing TNF-α immunoreactivity (red) and NeuN (green). Thee was an intense increase in TNF-α expression in neurons, although thesource of such immunoreactivity might also be derived from other cellular sources (for example, microglia) or from the circulation viablood–brain barrier transport (see text).

#### Th1 cytokines in cortex

We examined changes in the expression of Th1 cytokines in the frontotemporal cortex. These experiments showed not only temporally dependent changes in gene expression of TNF-α (fold increase 2.23 ±0.25; *F* = 7.639; *P* < 0.004) (Figure 5A) and TNFR1a gene expression (fold increase 2.82 ±0.24; *F* = 14.312; *P* < 0.0001) but also in protein levels (88.30 ±39.42 in controls vs. 523.71 ±45.48 after 15 days of SF; *F* = 31.05; *P* < 0.001) (Figure 5B), the latter appearing to be preferentially localized in neurons within the cortex (Figure 5C). By contrast, no changes occurred in the gene expression of other cytokines, such as IL-1β and IL-6 (Figure 5).

**Figure 6:**
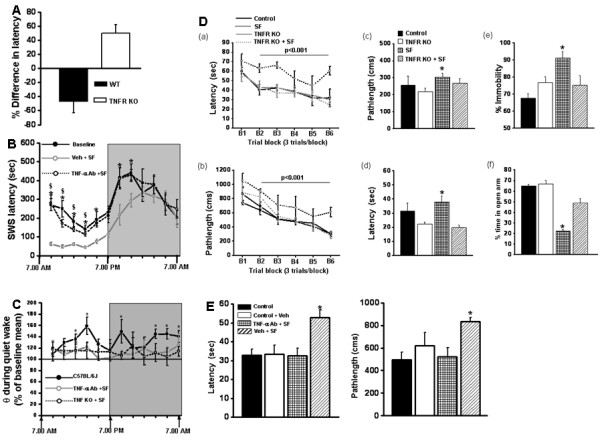
**Changes in sleep patterns in mice after sleep fragmentation (SF).** (**A**) Changes in mean sleep latency after 15 days of sleep fragmentation in 57BL6/J and tumor necrosis factor receptor (TNFR) knockout mice when compared with corresponding controls (n = 6;*P* <0.001). (**B**) Changes in mean slow wave sleep latency during the circadian cycle in mice exposed to SF for 15 days (open circles) and treated with vehicle (grey line) or TNF-α neutralizing antibodies (dotted line) and mice under control sleep conditions. (*P <0.01; n = 7/group). (**C**) Increases in theta frequency during quiet waking in C57BL/6 J mice, indicating = increased sleepiness after SF. (**D**) Mean latencies (seconds)and path lengths (cm) to locate the target platform during (**a, b**) place training, (**c, d**) reference memory after training, and (**e, f**) immobility in the forced swim test in C57BL6/J and TNFR knockout mice exposed to SF or control sleep conditions (*P <0.01; n = 12/group). (**E**) Mean latencies(seconds) and path lengths (cm) to locate the target platform during place training in mice exposed to SF or control sleep conditions, and treated with vehicle or TNF-α neutralizing antibodies. (*P <0.01; n = 12/group).

### Sleep architecture and behavioral changes in tumor necrosis factor-α receptor knockout mice after 15 days of sleep fragmentation

#### Measurements of wake and sleep

EEG monitoring of TNFR-KO mice during 12 hours of LP showed that they were awake for 34.71 ± 1.14% of the time without SF, which increased to 42.66 ± 0.85% after 15 days of SF (*P* < 0.05). During DP, these percentage were 62.13 ± 0.81% and 56.56 ± 0.74%, respectively (*P* < 0.05). The percentage of time the TNFR-KO mice spent in SWS was 58.1 ± 1.07% before SF and 56.09 ± 1.13 after 15 days of SF (*P* < 0.05) for LP, and 34.64 ± 0.87% and 36.9 ± 0.66% for DP (*P* < 0.05). The time spent in REM was 7.68 ± 0.77% before SF and 6.6 ± 0.44% after SF during LP, and 3.47 ± 0.88% and 4.83 ± 0.13, respectively, during DP (p < 0.05). There were no changes in time spent in wake, SWS and REM sleep during the 24 hour period. After 15 days of SF, SWS latency was markedly reduced in the wild-type controls compared with the TNFR-KO mice (−46.68 ±16.80% and +49.73 ±12.32, repectively; *F* = 18.823, *P* < 0.001) (Figure 6A). On day 15. the percentage of delta frequency during DP was non-significant in C57BL/6 J and TNFR-KO. Although the KO mice exhibited significantly higher absolute delta power than the C57BL/6 J mice [*P* < 0.001), the percentage change from baseline mean showed only a trend towards increase in delta.

#### Spatial learning performance

On a standard place discrimination task, a new group of C57BL6/J mice exposed to 15 days of SF (SF-C57BL6/J) exhibited longer latencies and path lengths to locate the hidden platform compared with the sleep controls (SC- C57BL6/J) or the SC-TNFR-KO or SF-TNF-KO mice (that is, without and with SF, respectively) (Figure 66Da, Db). Overall latency analysis for the entire trial blocks showed significant changes between the different treatment groups for latency (*F* = 6.817; *P* < 0.001) and path length, (*F* = 8.192; *P* < 0.001), indicating that SF adversely affected task performance for C57BL6/J mice only. Significant differences in latencies were seen during blocks 2 (*F* = 3.519; *P* < 0.003), 3 (*F* = 8.39; *P* < 0.001), 4 [F = 3.35; *P* < 0.03) and 6 (*F* = 20.14; *P* < 0.001). There were no significant differences in blocks 1 or 5. Repeated measures ANOVA showed significant differences in path lengths during block 6 (*F* = 38.60; *P* < 0.001), with no significant differences in other blocks. In the reference memory tests, SF-C57BL6/J mice exhibited significant deficits in memory retention in both latency (*F* = 7.943; *P* < 0.001) and path length (*F* = 1.657; *P* < 0.05). However, the SF-TNF-KO mice performed similarly to sleep controls (Figure 6Dc,e 6Dd). Repeated measures MANOVA with latency, groups, and conditions (*F* = 74.61; *P* < 0.0001) showed that SC TNF KO and SC C57BL6/J mice required significantly less time than their SF littermates to find the hidden platform in a Morris water maze (Figure 6Da); Repeated measures MANOVA with path length, groups, and conditions (*F* = 73.79; *P* < 0.0001) indicated that as the training progressed, the SC TNF KO and SC C57BL6/J mice reached the hidden platform and covered the shortest distance compared with their littermates exposed to SF in a Morris water maze (Figure 6Db).

#### Forced swim test and elevated plus maze

SF- C57BL6/J mice had significantly longer periods of immobility during the last 4 minutes of the FST (*F* = 3.73; *P* < 0.03) compared with all other treatment groups, including SF-TNF-KO mice (Figure 6De). Similarly, there were significant differences in the percentage of time SF- C57BL6/J mice spent in the open arm (*F* = 32.57; *P* < 0.001) (Figure 6Df) compared with the other three groups.

### Sleep architecture and behavioral changes after 15 days of treatment with a tumor necrosis factor-α neutralizing antibody

A 24-hour EEG analysis of mice injected with saline and subjected to SF for 15 days found comparable results to that of C57BL6/J mice subjected to SF alone (data not included). These mice were awake for similar lengths of time at baseline (34.53 ±2.62%) and after 15 days of TNF-α Ab + SF (34.83 ±0.39%), and the difference was not significant (*P* < 0.05). During the 12 hours of LP, untreated mice spent 57.27 ±2.7% of the time in SWS, which reduced significantly after 15 days of TNF-α Ab + SF (53.88 ±1.27%) (*P* < 0.02).A similar trend was also seen for REM sleep (8.19 ± 0.56% with saline vs 7.28 ±2.15% in TNF-α Ab + SF). There was no significant difference in SWS latencies between saline (−86.857 ±21.45%) and TNF-α Ab + SF (−96.53 ±18.9%) mice on day 1 during LP. On day 15 SF, the percentage difference in SWS latency was reduced in saline-treated mice (−223.84 ±35.09%) compared with mice treated with TNF-α Ab + SF (−21.39 ±18.19%, *P* < 0.001) (Figure 6B), indicating that TNF-α Ab injection prevented the SF-associated excessive sleepiness. During DP, immediately after cessation of the SF procedure, the latency to SWS showed a tendency to return to baseline levels (Figure 6B). On day 15, the percentage difference in SWS latency was further reduced in saline mice (−223.84 ±35.09%) compared with TNF-α Ab + SF (−21.39 ± 18.19%) (*P* < 0.001), indicating that TNF-α Ab injection prevented the SF-associated excessive sleepiness. Overall comparison of theta power during quiet wakefulness, a marker of sleepiness, showed a significant increase in C57BL/6 J after 15 days of SF (*F* = 1.450; *P* < 0.039) (Figure 6C). There was an increase in theta power during both LP and DP after 15 days of SF in the C57BL/6 J mice only (Figure 6C). Such changes were not seen in either the TNFR-KO or TNF-α-Ab injection groups after SF (Figure 6C).

### Retention after TNF-α neutralizing antibody injection

In the reference memory tests, SF-C57BL6/J mice injected with vehicle (saline) exhibited significant deficits in memory retention (Figure 2E) in both latency (*F* = 7.033; *P* < 0.001) and path length (*F* = 3.743; *P* < 0.016) compared with all the other groups, indicating that TNF-α Ab injection prevented SF-associated behavioral deficits.

## Discussion

In this study, we aimed to investigate the effects of SF on mice, and found that SF mimicking the recurrent arousals associated with sleep apnea induces both increased sleepiness and neurocognitive deficits. In addition, we sought to investigate the role of the TNF-α mediated pathway in SF and its relationship to measures of sleep architecture and cognitive behavior. In healthy people, serum inflammatory markers have been associated with abnormal sleep architecture [59]. Patients with obstructive sleep apnea show significant increases in serum levels of TNF-α, IL-1β, and IL-6 [60-64]. Because there is a scarcity of data on the association between EEG arousals and inflammation in patients with obstructive sleep apnea (OSA), the role of cytokines in disrupting the architecture of sleep will need further investigation. Of note, a study by Yue *et al*. [65] suggested that TNF-R1, but not TNF-α, is associated with arousals during sleep in patients with OSA.

Depressive and anxiety symptoms are common in patients with OSA [66-68], and in the present study, similar symptoms were reproduced in the model of chronic SF, suggesting that the recurrent arousals play a role in these symptoms. The EPM is the most frequently used apparatus for assessing anxiety-like behaviors in animals, [69,70] because it enables researchers to observe the conflict between two innate rodent behaviors: the avoidance of open space exposure, conflicting with the tendency to explore novel environments [70]. When placed in the EPM, naive mice will by nature tend to explore the open arms despite their natural fear of heights and open spaces. In the present study, our results further imply that SF modifies anxiety-like behaviors in C57BL/6 J mice.

### Sleep fragmentation and bioenergetics in brain

We found that, in contrast to SD, SF does not curtail sleep duration and also does not induce reductions in ATP or intracellular energy sources, as evidenced by the absence of AMPK phosphorylation after SF. However, the SF-induced cognitive and behavioral alterations were associated with reduced CREB phosphorylation and transcriptional activity in the hippocampus, and with increased expression of PDE4. Because sleep deprivation has been associated with reduced brain ATP levels and increased phosphorylation of AMPK [71-76], and with induced expression of Homer1a [77], we further sought to determine whether such changes in brain bioenergetics and gene expression would account for the SF-induced cognitive and behavioral phenotypes. ATP levels were unaltered by SF, and no evidence for increased AMP kinase-α phosphorylation or Homer1a expression occurred. This clearly showed that altered cellular bioenergetics did not underlie any of the phenotypic features associated with SF, namely, increased sleep propensity and neurobehavioral and cognitive dysfunction. Although the mechanistic link between SF and the putatively reduced bioavailability of cAMP will have to await further exploration, the now compellingly established role of CREB transcription in memory and learning definitely suggests that these pathways will be disrupted by upstream increases in TNF-α activity.

In the present study, we found that mice that are periodically awakened during their sleep period exhibit sustained increases in sleep propensity in the presence of globally preserved sleep patterns and duration. Indeed, sustained implementation of SF results in normalization of the total duration of sleep and waking, and in delta power. However, preserved sleep duration in the context of chronic SF is not accompanied by normal sleep latencies, thereby indicating increased sleepiness in the absence of sleep curtailment. This finding is further corroborated by the increases in theta frequency during quiet waking periods during the DP interval. In other words, normalization after long-term SF of the commonly used and widely accepted standard measures of sleep integrity did not abrogate the increases in sleepiness, suggesting that perturbations in sleep continuity play a crucial role in the maintenance of wake drive and of wake-related cognitive, mood, and behavioral tasks.

### Sleep fragmentation increases TNF-α in brain

Based on previous evidence suggesting that TNF-α has important regulatory effects on sleep, and that TNF-α plays a role in the occurrence of excessive daytime sleepiness in children who have disrupted sleep as a result of OSA, a condition associated with prominent SF [78-82], we explored several Th1 cytokines. However, it should be noted that a recent study in adults with sleep apnea failed to identify an association between polysomnographic variables and serum TNF-α levels, even though the frequency of respiratory-induced arousals was associated with the concentrations of soluble TNFR1 in these patients [65]. Of the Th1 cytokines explored in this study, only increases in TNF-α were seen. Concordant with such findings, we then subjected TNFR-KO to SF, which elicited similar increases in cortical TNF-α tissue levels (data not shown); however, these were not accompanied by any significant changes in sleep propensity or in cognitive function. As a corollary to such findings, the source of TNF-α was not identified, and could originate from multiple sources, such as microglia, neurons, or even from the peripheral circulation via regulated transporters in the blood–brain barrier [83,84]. Indeed, systemic treatment of C57BL6/J mice with a neutralizing TNF-α antibody during the course of SF also prevented SF-induced increases in sleepiness and cognitive deficits. Thus, although SF probably activated sleep homeostatic responses that ultimately resulted in preserved overall sleep duration and characteristics, a release occurs of biological mediators with somnogenic properties, such as TNF-α, and this is accompanied by detrimental effects on hippocampal task performance, similar to those seen after sleep deprivation [85,86]. We further summarize that these behavioral changes could also reflect altered cAMP signaling, particularly considering the increased expression levels of PDE4 that occur after prolonged SF and the reductions in phosphorylated CREB in the cortex and hippocampus of SF-exposed mice [87]. The chronic SF-induced phenotype is also reminiscent of the anxiety-like behaviors induced by disrupted sleep patterns in the context of light exposure during sleep [88].

**Figure 7:**
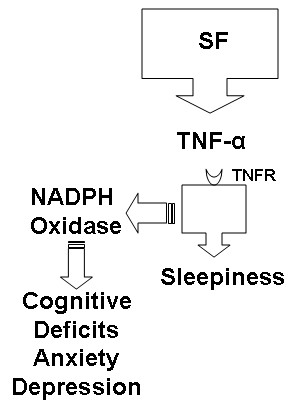
**Putative schematic diagram linking sleep fragmentation (SF) to increased activation of pathways medicated by tumor necrosis factor (TNF-α) and those mediated by NADPH oxidase (see main text and Nair*et al***[48]**for more details)**.

Although these findings are essentially confirmatory of our previous study [48], we further expanded the scope of this work in an attempt to unravel the potential mechanisms underlying sleepiness in this model, because genetic ablation of NADPH oxidase prevented cognitive deficits but failed to abrogate the reductions in SWS latencies [48]. We now propose that SF induces sleepiness via initial and rather selective activation of TNF-α pathways, which then will activate an NADPH-dependent oxidative stress cascade that will ultimately lead to cognitive dysfunction along with anxiety-like behaviors and depression (Figure 7).

## Conclusions

A major challenge in the field of sleep research has been to determine how sleep disruptions resulting from an extensive list of perturbations included in everyday living, or those induced by illnesses such as sleep apnea, can affect cognitive function and mood. The findings presented here define a crucial role for TNF-α in this context, and further support the link between sleep and immunity [89], even in the absence of sleep curtailment or ATP deficits [90]. Our data suggest that if compounds can be developed to block the activity or expression of TNF-α, or to selectively target TNF-α pathways in brain structures, they may prove useful in the treatment of the cognitive and mood effects of sleep disruption.

## Abbreviations

AMP, Adenosine monophosphate; ANOVA, Analysis of variance; ATP, Adenosine triphosphate; BL, Baseline; CREB, Cyclic AMP response element-binding protein; DP, Dark period; EEG, Electroencephalogram; EMG, Electromyogram; EPM, Elevated plus maze; FFT, Fast Fourier transform; IL-1β, Interleukin-1beta; I-L6, Interleukin-6; LP, Light period; LTP, Long-term potentiation; MANOVA, Multivariate analysis of variance; NADPH, Nicotinamide adenine dinucleotide phosphate; PDE, Phosphodiesterase; REM, Rapid eye movement; SF, Sleep fragmentation; TNFR KO, TNF receptor double knockout mice; TNF-α, Tumor necrosis factor-alpha.

## Competing interests

The authors have no competing interest to declare in relation to this manuscript.

## Authors’ contributions

DG and YW conceived and designed the study;VR, DN, SZ, FH, NK, and DG were responsible for analysis and interpretation;, and VR, DN and DG for drafting the manuscript for important intellectual content. VR, DN, SZ, NK, FH, FK, RL, AC took part in the experimental procedures. All authors read and approved the final manuscript.
